# Health Sciences Students: Witnesses of Discrimination in the Care of Foreign Patients in Chile

**DOI:** 10.17533/udea.iee.v43n1e09

**Published:** 2025-04-28

**Authors:** Debbie Jeinnisse Álvarez-Cruces, Assumpta Aneas Álvarez, Alejandra Nocetti-de-la-Barra, Juan Mansilla Sepúlveda

**Affiliations:** 1 Nurse and Dental Surgeon, PhD. Associate Professor, Universidad de Concepción, Chile. Email: debbiejalvarez@udec.cl. Corresponding author. https://orcid.org/0000-0003-0652-000X Universidad de Concepción Universidad de Concepción Chile debbiejalvarez@udec.cl; 2 Pedagogist, PhD. Full Professor, Universidad de Barcelona, Spain. Email: aaneas@ub.edu. https://orcid.org/0000-0002-9519-6696 Universitat de Barcelona Universidad de Barcelona Spain aaneas@ub.edu; 3 Biology Professor, PhD. Associate Professor, Universidad Católica de la Santísima Concepción, Chile. Email: anocetti@ucsc.cl. https://orcid.org/0000-0003-2509-8051 Universidad Católica de la Santísima Concepción Universidad Católica de la Santísima Concepción Chile anocetti@ucsc.cl; 4 Professor of History, Geography, and Civic Education, PhD. Associate Professor, Universidad Católica de Temuco, Chile. Email: jmansilla@uct.cl. https://orcid.org/0000-0001-8175-7475 Universidad Católica de Temuco Universidad Católica de Temuco Chile jmansilla@uct.cl

**Keywords:** professional training, Health Sciences, disparities in health care, perceived discrimination, migrants, formación profesional, ciencias de la salud, disparidades en atención de salud, discriminación percibida, migrantes., formação profissional, ciências da saúde, disparidades em assistência à saúde, discriminação percebida, migrantes.

## Abstract

**Objective.:**

This work sought to inquire on the perception of Health Sciences students about the health care provided to patients of foreign origin in clinical environments.

**Methods.:**

Phenomenological approach with multiple case study design. Health Sciences students from three Chilean universities participated with intensity sampling. Semi-structured interviews were conducted via Zoom after the consent was signed. Data analysis included open and axial coding axial with the aid of the ATLAS.ti 24 software.

**Results.:**

The study had the participation of 106 students, who identified different types of discrimination, besides diverse exclusion manifestations that take place during the care process, such as: apathy, prejudice/stereotyping, derogatory comments, evading, underestimating, normalizing discrimination, infantilizing, and scoffing. Some of the participants normalized these behaviors due to the overload health system.

**Conclusion.:**

The Health Sciences students witnessed several types of discrimination and exclusion manifestations occurring during the care process, highlighting apathy and evading as characteristic of the clinical environment, which could favor negative vicarious learning that perpetuates discriminatory conducts against foreign patients.

## Resumen

**Objetivo.:**

Indagar en la percepción del estudiantado de Ciencias de la Salud sobre la atención sanitaria proporcionada a los pacientes de origen extranjero en los ambientes clínicos.

**Métodos.:**

Enfoque fenomenológico con diseño Estudio de Caso Múltiple. Participaron estudiantes de Ciencias de la Salud de tres universidades chilenas seleccionados con muestreo por intensidad. Se realizaron entrevistas semiestructuradas por Zoom luego de firmar el consentimiento. El análisis de los datos incluyó codificación abierta y axial con la asistencia del software ATLAS.ti 24.

**Resultados.:**

Participaron 106 estudiantes, quienes identificaron diferentes tipos de discriminación, además de diversas manifestaciones de exclusión que ocurren durante el proceso de atención, como: desidia, prejuicio/estereotipo, comentarios despectivos, eludir, menospreciar, normalizar la discriminación, infantilizar y burlarse. Algunos de los participantes normalizaron estos comportamientos debido a la sobrecarga del sistema sanitario.

**Conclusión.:**

El estudiantado de Ciencias de la Salud fue testigo de varios tipos de discriminación y manifestaciones de exclusión que ocurren durante el proceso de atención, destacaron la desidia y eludir como propias del ambiente clínico, lo cual podría favorecer un aprendizaje vicario negativo que perpetúe conductas discriminatorias a pacientes extranjeros.

## Resumo

**Objetivo.:**

Investigar a percepção de estudantes de Ciências da Saúde sobre o atendimento à saúde de pacientes de origem estrangeira em ambientes clínicos.

**Métodos.:**

Abordagem fenomenológica com delineamento de estudo de caso múltiplo. Participaram estudantes de Ciências da Saúde de três universidades chilenas, selecionados por amostragem de intensidade. Entrevistas semiestruturadas foram conduzidas via Zoom após assinatura do termo de consentimento. A análise de dados incluiu codificação aberta e axial com o auxílio do software ATLAS.ti 24.

**Resultados.:**

Participaram 106 estudantes, que identificaram diferentes tipos de discriminação, bem como diversas manifestações de exclusão que ocorrem durante o processo de cuidado, tais como: apatia, preconceito/estereótipo, comentários depreciativos, evitação, menosprezo, discriminação normalizadora, infantilização e deboche. Alguns participantes normalizaram esses comportamentos devido à sobrecarga do sistema de saúde.

**Conclusão.:**

Os estudantes de Ciências da Saúde testemunharam vários tipos de discriminação e manifestações de exclusão que ocorrem durante o processo de cuidado, destacando a apatia e a evitação como típicas do ambiente clínico, o que pode favorecer a aprendizagem vicária negativa que perpetua o comportamento discriminatório em relação aos pacientes estrangeiros.

## Introduction

In health training, ethical and moral commitment is promoted to practice the profession without distinctions, focused on respect and the intrinsic value of each person.^1^ In addition, the importance of respecting the duties and rights of patients throughout the care process is emphasized. However, scientific evidence indicates implicit discrimination or micro-discrimination that even the person is not able to perceive because they are unaware of such.^2^^,^^3^ Besides, the health system is often overwhelmed by high demand, which generates burnout and exhaustion among health staff.^4^These work conditions can generate two situations: that the foreign population perceives the system’s deficiencies as discrimination or that the staff justifies poor attention due to the fatigue and stress they endure. Objective 17 of the Global Compact for Migration states that “all forms of discrimination must be eliminated and evidence-based public discourse promoted to change perceptions of migration.”^5^ Consequently, evidence is needed to highlight and expose manifestations of exclusion that occur in health care against the foreign population, which could represent discriminatory acts.

Literature from different parts of the world reports that, in health care, discrimination situations take place, such as: xenophobia, classism, and racism against the foreign population, especially against those considered of lower category,^2^^,^^3^ a situation that is not different in Chile.^6^ Notwithstanding, the manifestations that denote a discriminatory component in health care are unknown. Discrimination implies any exclusion or different treatment to a person product of their origin, gender, religion, age, health condition, or any other situation. Although anyone can be object of discrimination, it is observed that those who endure any disadvantage are those who suffer from it for a longer period of time and more frequently.^3^ In the clinical setting, research has centered on knowing the foreign population’s perception on the health care received^7^^,^^8^ and the challenges health professionals have had to face to provide health care to foreigners.^9^^,^^10^

The literature reports that medical students diminish their favorable attitudes, altruism, responsibility, and social commitment towards foreigners as they progress through their training, unlike those students who belong to minority groups or who were born in another country.^11^ Nursing students report a high score in items of awareness, knowledge, and respect in different questionnaires applied; however, negative attitudes are maintained towards refugees due to factors associated with the patient’s gender, age, financial conditions, educational level, and religion despite having intercultural content in their professional training.^12^^,^^13^ Although qualitative research is recommended to analyze manifestations of discrimination towards foreign patients, research in this field is scarce. One such research analyzes the nature of relationships of empathy, comprehension, and commitment Health Sciences students have when providing care to said group of patients.^14^ Another study identified self-management strategies for student competencies, which included respect and comprehension toward the foreign population object of care, despite having had negative modeling by clinical guides or other health professionals who may have carried out discriminatory acts during their professional and teaching practice.^15^ Likewise, a recent research indicates that the students observed racism omnipresent in health care, in addition to poor coping strategies when they identified it.^16^ This greater sensitivity, openness, and disposition towards disadvantaged groups would be related to the individual's self-reflective capacity.^15^^,^^17^

 Based on the foregoing, this research was proposed from a qualitative approach to inquire on the perceptions of Health Sciences students about the health care provided to patients of foreign origin in clinical environments.

## Methods

This was a phenomenological qualitative study with multiple case study design, which is characterized by analyzing two or more cases on a particular topic because they share common characteristics; however, they differ in some aspects.^18^ Thus, three geographically distant universities from Chile were included. The selection of participants was conducted via intensity sampling, so students in the final stage of training or in professional internship in the careers of medicine, midwife, nursing, nutrition, dentistry, pharmacy and kinesiology were selected. The study was approved by two Scientific Ethics Committees accredited in Chile, besides the ethics committees in the university institutions that were part of the research. 

A mass invitation, with the informed consent attached, was sent by email to all students. It detailed the objective of the research and the scope the results could have in benefit of improved training of future health professionals. This consent had to be signed prior to scheduling the interview.

The information collection strategy involved a semi-structured interview with eight open-ended questions. This allowed participants to freely express their experiences, appreciations, and evaluations regarding the health care provided to foreign patients and the presence or absence of discrimination. To avoid biasing the participants' responses, the interview began with the following questions: 1- What have been your experiences with foreign patients in health care? 2- How do you feel about the care provided to foreign patients by other members of the health team? 3- Have you observed differences between the care provided to Chilean and foreign patients? 4- During care, was cultural influence on the health/disease process considered? 5- How do you rate the care you provided to foreign patients? Why? If any of the prior questions indicated any discriminatory component against foreign patients in health care, the following questioning was continued: 6- What type of discrimination did you observe during the care process against foreign patients? 7- What attitudes or behaviors do you consider discriminatory against foreign patients in health care? 8- According to your perception, how could care be improved towards foreign patients?

The interviews lasted between 40 and 90 minutes and were conducted via the Zoom platform by the principal researchers, who holds a PhD with post-graduate training in health and education. The files were stored in audio format (MP3) to safeguard the participants’ identities. The data analysis included open and axial coding. The first consisted of a deductive phase to identify the types of discrimination reported by the literature, which became categories. Subsequently, a second inductive analysis was conducted to examine the situations reported by the participants that could represent behaviors, attitudes, or circumstances of exclusion in the care of migrants. Through this reflexive and recursive analysis, subcategories were obtained that corresponded to manifestations of discrimination that take place during the care process towards foreign patients. Using the ATLAS.ti 24 software, during axial coding, the types of discrimination were related to the various manifestations perceived to delve deeper into the new forms in which discrimination occurs in health care. 

To present the results, the Sankey diagram was preferable over the co-occurrence table, which only provides numerical values. Said diagram evidences in a clearer and more explanatory way the density of the codes and the relationship among them, permitting greater comprehension. During the research, the principles of scientific rigor were upheld. Thus, credibility and confirmability were achieved with an extensive period of data collection to capture the different perspectives on the phenomenon under study, which were subject to the judgment of the informants. In addition to the above, data saturation and triangulation by career and university were achieved. This allowed for an adequate analysis and interpretation of the data, supported by direct quotes from the participants. 

Transferability was achieved by including participants from different careers and universities who shared the common experience of caring for migrant patients during their professional training. Dependency was established by first analyzing the types of discrimination reported in the literature, then investigating the behaviors and attitudes that represented exclusionary manifestations in the care of foreign patients. These findings were subsequently reviewed by the research team. 

### Results

The study included 106 students, comprising 35 men and 71 women from various health careers and universities (Table 1). The highest proportion of participants came from University 2, which offers all the health careers included in this study, followed by University 1 and University 3.

**Table 1:** Characterization of participants

	University 1			University 2			University 3		Total
Career	Man	Woman	Man	Woman	Man	Woman	
Medicine	4	5	7	1	NA	NA	17
Midwife	NA	NA	2	7	0	1	10
Nursing	2	5	2	6	0	1	16
Nutrition	1	7	0	8	0	6	22
Odontology	NA	NA	5	5	NA	NA	10
Pharmacy	NA	NA	5	5	NA	NA	10
Kinesiology	3	4	1	7	3	3	21
Total	10	21	22	39	3	11	106

NA: not applicable. The career is not taught in the university

From an overall approach, when asked directly whether they had observed discriminatory attitudes from health staff while caring for foreign patients, 64 of the 106 participants reported having witnessed discrimination, the remaining 42 indicated not having witnessed discrimination during health care; nevertheless, they declared that it occurred during the moment before or after the clinical care, that is, it was hidden. So, practically all the participants indicated having observed discriminatory conducts at some point during the health care process. 

Another relevant result was that discrimination was observed as a transversal phenomenon, given that it took place in all care settings and professional profiles. Furthermore, Students noted that the older health staff (> 55 years of age) were those who displayed the most discriminatory behavior with their patients and users. 

From the deductive analysis, six categories were established corresponding to types of discrimination existing in the scientific literature consulted: a) relationship of power, b) structural, c) racism, d) xenophobia, e) micro-discrimination, and f) aporophobia. Table 2 provides an operational description of each of these categories, the frequency of associated quotes, and the direct quote that supports it. 

**Table 2:** Types of discrimination, operational definition, frequency, and direct quote

Type of discrimination	Operational definition	Quote freq.	Direct quote
Relationship of power	Situation in which the health staff feels superior to the foreign patient, whether due to nationality, knowledge, economic, political, educational situation, etc.	46	*“Simply because you are a foreigner: Venezuelan, Bolivian or any other nationality, whether from South America, regardless of your social class, you will be discriminated against in health care in Chile. You can have a thousand professional degrees, have an awesome job, but you will always be looked down upon. Whoever it is, it could be the doctor, nurse, etc., regardless of who it is, they will be looked down upon, so to speak. That is what I have noted lately, especially during this recent pandemic.” MMi1(2)*
Structural	Situation in which the health system does not offer appropriate conditions to provide adequate care to foreign patients, for example: translator, forms, and signage in the patient's language, longer time in care, etc.	38	*“I mean, to start with, not having a translator, we're already starting off badly because, how are we going to care for them if they are Haitian and don't know Spanish? We will not know what they have, we won’t know what they feel, the symptoms, the only thing we could know would be the vital signs, the physical exam. So, the biggest problem is the language and that we are not provided with the means to communicate with the patients.” WNu2(1)*
Racism	Situation in which the health staff expresses attitudes or harmful behaviors product of the foreign patient’s skin color	35	*“Even because of the skin color, for example, when they arrive with dermatological conditions. Because the patient has black skin, it is not possible to see, for example, erythema or any other lesion, so the patient is more complex. Then, generally, since they complain, they are made to wait or leave them for later. I don't know if they leave them at the end, but it's like the doctors are lazy or you are told to see them.” MM1(1)*
Xenophobia	Situation in which the health staff expresses attitudes or harmful behaviors product of the foreign patient’s nationality	26	*“Especially in situations where there is a collective rejection of the arrival of immigrants in general, but especially South Americans. I don’t know why they are so badly received in Chile. And, likewise, in the hospital, you notice the difference with the different types of comments made, which is not like explicit discrimination, but rather more subtle.” WMi6(2)*
Micro-discrimination	Situation in which the health staff, unconsciously, express attitudes of disapproval, undervaluation, condescension, or prejudice towards the foreign patient	21	*“Let’s see, I don’t think there is a negative intention, no. But the problem, I believe, we have, as Chileans, is that we normalize the mockery, which - in the end - are no joke (…) But that is now a problem we have as society in general, in every sense.” MN4(1)*
Aporophobia	Situation in which the health staff expresses attitudes or harmful behaviors due to the foreign patient’s poverty situation	13	*“And also, sometimes, I saw a given type of prejudice. It was like they saw people with a certain appearance and they would leave them aside. It's like the typical prejudices we have: 'No, this person who looks better dressed, let's give preference to this one'.” WP10(2)*

Coding of the direct quotes was conducted in the following manner: First letter: participant’s sex (man: M and woman: W), second letter: participant’s career (medicine: M, nursing: N, midwife: Mi, pharmacy: P, kinesiology: K, odontology: O, and nutrition: Nu), number without parenthesis: number designated to the participant according to the career, number with parenthesis: number designated to the university. 

**Figure 1:**
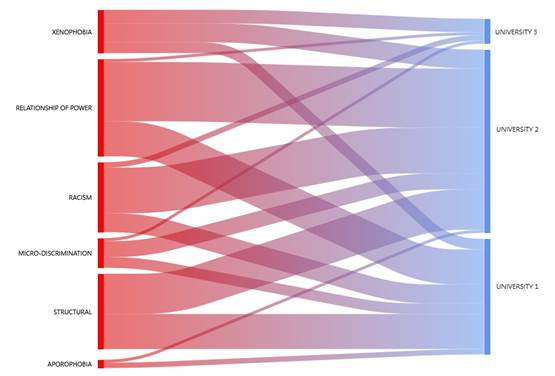
Types of discrimination according to university institution

Using the ATLAS.ti 24 program, comparison was made among the three universities, included in this study, with the types of discrimination reported. Figure 1 shows that students from Universities 1 and 2 witnessed all the types of discrimination mentioned in Table 2; unlike University 3, which only reported xenophobia, racism, micro-discrimination, and relationship of power. Students from University 1 reported more structural discrimination and relationship of power; University 2, relationship of power, racism, and structural discrimination; and University 3, xenophobia.

From the inductive analysis of the text, a series of subcategories emerged that allowed identifying various behaviors, attitudes, or circumstances considered exclusion manifestations, which had other modes, nuances, and levels in the expression of discrimination, different from those reported by the literature. These were: a) apathy, b) prejudice/stereotyping, c) derogatory comments, d) evading, e) underestimating, f) normalizing discrimination, g) infantilizing, and h) scoffing. It can be noted how said displays occupy diverse ranges in the behavioral expression, the emotional charge, and the symbolic medium of aggression. The operational definition, frequency, and direct quote of the different manifestations of discrimination are detailed in Table 3.

**Table 3:** Manifestations of discrimination, operational definition, frequency, and direct quote

Manifestation of discrimination	Operational definition	Quote freq.	Direct quote
Apathy	Situation in which health care is conducted without considering the necessary adjustments to provide care that considers culture, language, customs, beliefs or other of foreign patients.	79	[While caring for a Haitian patient] *“She [the tutor] was teaching her about the nutrition for her child at 6 months. She had a pamphlet, but it was in Spanish. So, she was crossing out what the child should not eat. The mother did not understand much, but when she did not understand, she would look at her mom [grandmother] who spoke a bit more Spanish. Perhaps she should have provided more visual instructions, but she limited herself to reading the pamphlet in Spanish. Then there was a cultural difference, because the grandmother said: ‘we don’t eat that.’ So, it was difficult. She was given the recommendations, but if they weren’t used to Chilean food, most likely they wouldn’t do it. But she focused on education with the pamphlet in Spanish and then gave it to them. I think it would have been better to have some more educational material or in their language, for better understanding.” WNu6(1)*
Prejudice/ Stereotype	Situation in which the health staff has negative preconceptions about foreign patients	60	*“I remember the kinesiologist who was in charge of me, his comment was: 'I have nothing against immigrants, but it seems that in the end they are going to take our jobs from us, because they work for less money'. And I don't know, I think they can even be better professionals than us. I have also heard, several times, they say 'at the borders we have to be more careful because people come with diseases; they bring other microorganisms that don't exist here'. So, one has a very different vision, perhaps, than that of older people.” WK6(1)*
Derogatory comments	Situation in which the health staff expresses derogatory opinions about foreign patients	53	[Caring for Haitian patient] “*When we finished the treatment, I remember one of the professors there saying: ‘black mouth, big teeth, that’s going to be a drama for the extraction later’. I was blank, it was strong, I couldn’t get any words out, because everything coming out of this mouth could have consequences later, so I just kept quiet and looked down.” WO8(2)*
Evading	Situation in which health care to foreign patients is postponed or delegated due to fatigue, listlessness, apathy, laziness, etc.	42	*“But no, it's not like they treat them badly, no. The truth is that I haven't seen any degree of discrimination or abuse, but yes, perhaps, there is a certain refusal to care for them, as if they are not as motivated. Perhaps because they encounter a greater communication barrier, it will be a bit more difficult for them to get care, that may be why (…) They say, for example: ‘oh, a Haitian patient arrived, ‘Oh, okay, but you take care of him.’ It's like they pass the ball back and forth, to put it bluntly.” WN7(2)*
Underestimating	Situation in which the health staff minimizes the symptoms or does not consider the foreign patient's belief in care	40	*“I heard, as a hallway comment, that Venezuelans were whinier, as were Haitians, but with an annoying tone. And during labor and post-partum, comments were made in more derogatory manner, especially with Haitian women, as if they should not be taken into account when complaining because they were exaggerating their pain. So, it seemed to bother them [the health staff] that they complained so much.” WMi1(3)*
Normalizing discrimination	Situation in which the health staff has normalized derogatory treatment towards foreign patients. Generally using diminutives to mitigate them	29	*“I think that the thing about the ‘chinito’ and the ‘negrito’ will never be ousted from Chilean idiosyncrasy. Personally, it irks me because it's as if we were going around saying about Chilean patients, the ugly patient, the lame one, the short one, etc. So, we have a name for a reason, right? Or, finally, don't say 'the little black one, say 'the patient in bed 15/2' and that's it, like any other patient.” WNu3(3)*
Infantilizing	Situation in which the health staff disciplines foreign patients for behaviors they consider inappropriate (as a father/mother would do with a child)	22	[Haitian patient after the ward] *“So I asked [the patient] if she had wanted to urinate or maybe if she had wet the bed and she said: ‘it seems so’. So, I notified the nursing aide to go help change the bedsheets. And she began to taunt her, she started to question she did that, that it wasn't right, that it was for dirty people and a whole bunch of things that weren't right. I found it very horrible, honestly.” WMi4(2)*
Scoffing	Situation in which the health staff make derogatory jokes to foreign patients.	19	*“What I have seen are a lot of jokes (scoffing), especially among older doctors. For example, to Haitian patients, they say: ‘Hey! Sell me a Super 8’ or the typical phrase ‘2 for 300 or 5 for 500’, as if alluding to the fact that Super 8 is sold* ^(*)^ *. But, generally, it’s the older doctors and who do have these behaviors at every level, that is, with Peruvians, Bolivians, even with Chileans, with impoverished people in general.” MM1(1)* (*)In Chile, working conditions for irregular migrants are complex, so they opt for selling sweets on street corners to have daily sustenance, one of them is the so-called Super 8 (wafer cookie covered in chocolate).

Coding of the direct quotes was conducted in the following manner: First letter: participant’s sex (man: M and woman: W), second letter: participant’s career (medicine: M, nursing: N, midwife: M, pharmacy: P, kinesiology: K, odontology: O, and nutrition: Nu), number without parenthesis: number assigned to the participant according to the career, number with parenthesis: number assigned to the university.

**Figure 2:**
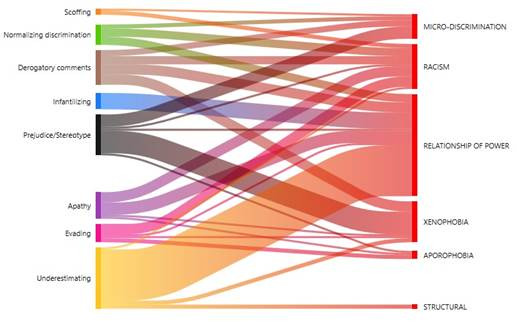
Co-occurrence analysis between type of discrimination with manifestations observed by students

The study proceeded to relate the types of discrimination with the manifestations discovered in this research. The Sankey diagram permitted visualizing that the highest density of codes was for the discrimination relationship of power, which is connected with all the manifestations described in Table 3; except for scoffing. It is followed by racism that mainly has manifestations of evading, apathy, derogatory comments, normalizing discrimination, scoffing, prejudice/stereotyping, and underestimating; and xenophobia with prejudice/stereotyping, derogatory comments, underestimating, evading, and apathy. (Figure 2). 

**Figure 3:**
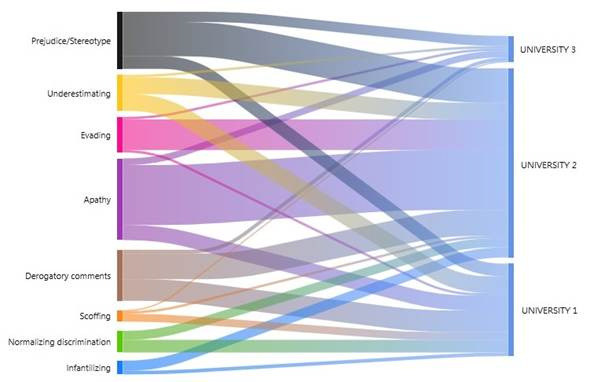
Manifestations of discrimination by University Institution

Similarly, a comparison was made of the students from the three universities regarding the different manifestations. It was highlighted that students from Universities 1 and 2 observed all the forms of manifestations described in Table 3, unlike University 3, which did not report witnessing attitudes of normalizing discrimination and infantilizing. In turn, students from University 1 witnessed more derogatory comments and apathy; University 2, apathy and evading; and University 3, prejudice/stereotyping and apathy (Figure 3).

**Figure 4:**
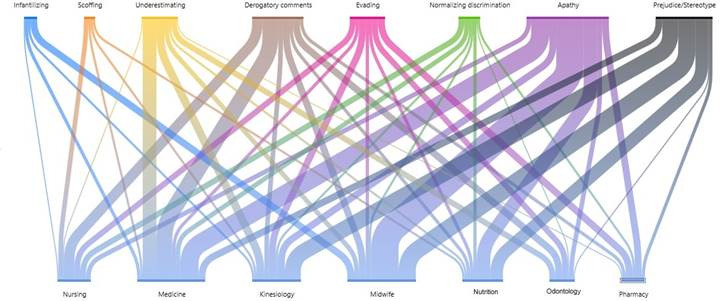
Manifestations of discrimination by career

The analysis by career permits noting that students observed different manifestations of discrimination. Nursing saw more derogatory comments; medicine, apathy, derogatory comments, and underestimating; kinesiology, prejudice/stereotyping; midwife and nutrition, apathy and prejudice/stereotyping; pharmacy, apathy and evading; and odontology, derogatory comments, apathy, evading, and underestimating. Furthermore, it is important to clarify that in the medicine and kinesiology courses, eight manifestations were observed; in nursing, midwife and nutrition, seven were observed, and in pharmacy and odontology, six (Figure 4).

**Figure 5:**
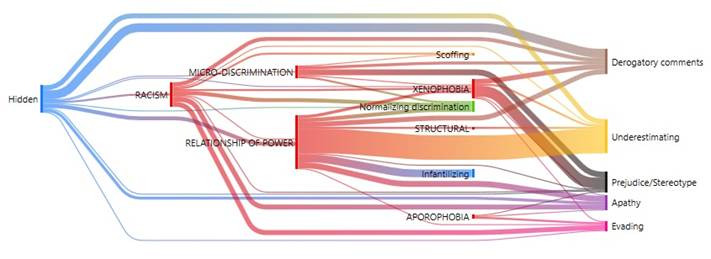
Relationship among types of discrimination with the different manifestations

Finally, different types of discrimination were related with diverse discrimination displays in health care (Figure 5). The Sankey diagram permits visualizing that the types of discrimination and their manifestations operated in hidden manner against the foreign population. Moreover, xenophobia, the relationship of power, and racism are linked with all the forms of manifestation that Health Sciences students indicate having witnessed when care was provided to foreign patients. 

## Discussion

The diverse clinical experiences the participants had during their professional training allowed their being witnesses to different types of discrimination in health care to foreign population reported in the literature. Among the typology used, the most recognized in the three universities studied was the relationship of power. This agrees with international studies,^2^^,^^3^^,^^19^ as well as with multiple investigations conducted in Chile, where a culture has been inherited that penalizes foreigners, especially South Americans, who are seen as inferior and unworthy of belonging to the country.^6^This research identified other conducts, attitudes, or circumstances valued as exclusion manifestations, which contributed certain nuances and degrees in discrimination. These were: a) apathy, b) prejudice/stereotyping, c) derogatory comments, d) evading, e) underestimating f) normalizing discrimination, g) infantilizing, and h) scoffing, which result in a relevant finding to understand how discrimination operates in the health environment and the subtleties it adopts.

In the co-occurrence analysis (Figure 2), it was interesting to note that the relationship of power was strongly linked to underestimating. This occurred, frequently, with foreigners with particular phenotypic characteristics, like darker skin color and unfavorable economic situation, which generate rejection by the Chilean population. This is also verified in international research, which report racial discrimination linked with the foreign patient’s socioeconomic condition.^16^^,^^20^^,^^21^ The manifestations most frequently reported by the different careers, notwithstanding the university, were apathy, prejudice/stereotyping, derogatory comments, underestimating, and evading. Among them, apathy and evading were highlighted as characteristic of health care. Both denote lack of interest and lack of willingness to carry out health care on the part of the responsible staff who delegate this task to others, like the students or other more sensitive staff who take on this care. Therefore, it could be said that discrimination against foreign patients has a secondary victim, which are those people in a more-vulnerable hierarchical position and, hence, they must assume tasks and responsibilities that do not correspond to them. Thus, one could speak of direct and indirect victims of the discriminatory practices applied by health professionals in caring for foreign patients. 

The differences in the types of discrimination reported among the distinct universities could be due to the varying clinical fields and the territorial zones in which they are located. Nevertheless, it may have been because there were less participants from University 3, so there could be a lack of representativeness in the reports. Likewise, manifestations of normalization and infantilization were also not reported by this last group; in comparison to Universities 1 and 2 which witnessed all the forms of manifestation included in this study.

It is important to note how the relationship of power, followed by racism, xenophobia, and aporophobia are closely linked with apathy and evading. These exclusion manifestations were justified and normalized by a certain group of students due to the high workload faced by health staff; however, they are indirectly linked to structural discrimination through a display of contempt. This could indicate that, although structural barriers exist that the health system still does not manage, In certain health staff, there are underlying discriminatory components resulting from personal ideological conceptions that permeate the clinical environment, as indicated by other research.^22^^,^^23^

The types of discrimination and exclusion manifestations, seemingly, occurred unconsciously and thoughtlessly on the part of the professionals who engaged in such, given that they did not hide said behaviors from their students; however, these turn out evident for the participants from the different careers and universities included in this study. This constitutes a negative model in the teaching/learning process during health care to foreign patients, which could be a powerful reason why Intercultural Competence has not yet achieved the desired results in the professional practice of the health area. Diverse research reports discrimination against foreign population during clinical care, in which students in training are present, so it is admissible to postulate the idea that there could be a negative vicarious learning towards the care of foreign patients.^11^^,^^13^^,^^21^^,^^24^Thereby, these results guide the way to approach the professional training of future Health Sciences students, with emphasis on coexistence, comprehension, respect, and development of otherness; unlike that observed until now, where the contents in Intercultural Competence focus on bio-epidemiological aspects and public health in the curriculum.^15^^,^^25^Nonetheless, a training process is indispensable designed with adequate pertinence, efficacy, effectiveness, coherence, and acceptance of others,^26^in addition to promoting critical reflection.^17^

Consequently, the training process must compromise, explicitly, the development of self-awareness to inquire on our characteristic prejudices and stereotypes that could influence the professional/patient relationship, the cultural identity to be aware of our patterns, beliefs, and customs influencing upon our decisions and behaviors; self-reflection to analyze the different experiences lived with patients to better manage feelings, attitudes, and knowledge; the critical capacity to deconstruct prevailing ways of thinking in favor of new more-inclusive perspectives; and learning to manage uncertainty to learn to face the new challenges that global society imposes with integrity, flexibility, and relevance. Likewise, the system, as a whole, must provide entities or organizations where discriminatory events can be reported, confidentially, but also offer information, guidance, training, and conflict mediation services, as other research has suggested.^20^^,^^27^In this sense, Nursing professionals play a key role in ensuring discrimination-free care management, which permits complying with the standards of inclusion, equity, and cultural pertinence, which the WHO and the PAHO have indicated. 

The greatest strength of this research is that it permits making visible the different exclusion manifestations that take place in the care process of foreign patients. Apathy and evading are even proposed as new forms of discrimination that occur in health care against the foreign population; unlike other types of manifestations that may be seen in any other setting of society. Therefore, the dissemination of these results is essential, both in the academic and clinical settings, because it may awareness for behavioral change and avoid their continued normalization and perpetuation, unconsciously, in the health staff. 

One of the limitations of this research is the low number of applications received from University 3, from northern Chile. Despite multiple e-mails, phone calls, and diverse invitations made by the institution and its academics, during 10 months, nobody else was registered to conduct the interview. Nevertheless, The results obtained were quite similar because they had also witnessed discriminatory practices against the foreign population. 

Future lines of research should be aimed at observational studies, which can account for more manifestations, which include interpretation of non-verbal language and proxemics, besides the characteristics of health professionals who have discriminatory practices, for greater comprehension of the phenomenon and intervene in a precise manner to benefit care with equity, inclusion and quality for the foreign population.

Conclusion. Health Sciences students who participated in this research witnessed different types of discrimination against the foreign population in health care. However, these discriminations did not always occur explicitly, but were hidden and/or adopted subtle displays, highlighting among them apathy and evading as characteristics of the health environment. It is cause for concern that certain participants normalize these attitudes due to the overload of the health system, which could indicate their perpetuating these practices in their future professional practice. Thereby, the training process of Health Sciences students must ensure the development of self-awareness as a foundation for getting rid of biases, prejudices, and stereotypes against the foreign population that are the basis of discrimination, in addition to self-reflection and critical capacity to understand how life experiences, beliefs, habits, patterns of coexistence and culture can influence the health decisions of foreign patients. Likewise, the health system must provide mediation units where students and foreign patients can go in case of needing help or guidance regarding any discrimination taking place during the care process.
